# The interactions between climate and soil factors influence the taxonomic and phylogenetic diversity of woody plants in the subtropical karst region

**DOI:** 10.7717/peerj.21365

**Published:** 2026-05-22

**Authors:** Rundi Zhou, Wei Shi, Yin Yi, Yao Wu, Xiaoxin Tang, Yaling Mao, Xiangrui Wang

**Affiliations:** 1 Key Laboratory of National Forestry and Grassland Administration on Biodiversity Conservation in Karst Mountainous Areas of Southwest China, Guizhou Normal University, Guiyang, China; 2 School of Life Sciences, Guizhou Normal University, Guiyang, China; 3 Engineering Research Center of Carbon Neutrality in Karst Areas, Ministry of Education, Guizhou Normal University, Guiyang, China

**Keywords:** Climate, Soil, Interaction, Taxonomic diversity, Phylogenetic diversity

## Abstract

The woody plants in subtropical forests are one of the most important long-term carbon sinks in the terrestrial ecosystems. However, its diversity patterns and the underlying mechanisms remain unclear. To explore the diversity patterns of woody plants in subtropical forests, based on the census data of 180 forest-dynamics monitoring plots in Guizhou province, this study assessed the effects of climate and soil factors, and their interactions on taxonomic and phylogenetic diversity using the negative binomial regression model. The results indicated that both climate and soil factors substantially improved the model’s prediction accuracy (model d). When interactions between climate and soil factors were further considered, explanatory power increased by 45.49% for taxonomic diversity (*R*^2^ = 0.774) and by 26.62% for phylogenetic diversity (*R*^2^ = 0.742) compared with the case without interactions. In particular, although soil factors such as total potassium (TK) had relatively weak direct effects, their interactions with climatic variables (*e.g.*, precipitation of the wettest month, Bio13) exerted substantially stronger influences. These interactions indicate that soil nutrient availability modulates plant responses to climatic variability, especially under conditions of fluctuating water supply. This may be attributed to the role of potassium in regulating stomatal function and enhancing plant responses to water availability under variable precipitation conditions. This study emphasizes the importance of environmental factors and their interactions in diversity patterns and community assembly of subtropical forests, which can provide empirical support for a deeper understanding of subtropical forest diversity patterns and assembly mechanisms.

## Introduction

As the principal components of forest ecosystems, woody plants serve as the main contributors to energy fixation and organic matter production. Their carbon sequestration and storage functions play a vital role in mitigating global climate change. Moreover, through the complex three-dimensional structures formed by the canopy, trunk, and understory layers, woody plants provide diverse ecological niches and stable habitats for animals and microorganisms with different ecological requirements, which underpin the forest microenvironments and support the integrity and biodiversity of the ecosystem (De Frenne et al., 2019). Given their central role in forest communities, understanding the spatial distribution patterns of woody plant diversity and the mechanisms driving these patterns has become a major focus in ecological research (Venter, Cramer & Hawkins, 2018; Zhang et al., 2020; Medeiros et al., 2025). The habitat filtering hypothesis posits that environmental conditions act as selective filters, determining which species from the regional species pool are able to persist and establish local communities (Zobel, 2016). Species with greater ecological similarity tend to occur under similar environmental conditions (Page & Shanker, 2018). However, plant colonization, survival, and growth are influenced not by a single factor but by the combined effects of multiple environmental variables. The direct, indirect, and interactive effects of these factors collectively regulate species growth, distribution, and environmental adaptability, thereby shaping the spatial structure of plant communities and determining both taxonomic and phylogenetic diversity patterns (Inague, Zwiener & Marques, 2021).

Climate and soil are critical factors that directly influence plant survival, growth, and distribution, and have been extensively studied to assess their impact on plant diversity patterns (Aguirre-Gutiérrez et al., 2020; Shi et al., 2020). However, the deterministic processes that shape diversity patterns may vary across spatial scales. Soil physicochemical heterogeneity typically plays a more significant role at finer spatial scales (Valencia et al., 2022), while climate factors tend to have greater influence at broader spatial scales. When considering the combined effects of soil and climate factors, their influence becomes more complex (Dattaraja et al., 2018).

Many previous studies have focused on the taxonomic diversity of plant species. However, integrating phylogenetic information into diversity research may offer deeper insights into community assembly mechanisms (Qin et al., 2020). Species richness (SR), a widely used index of taxonomic diversity, is directly related to biomass, nutrient cycling, and other ecological processes. The phylogenetic diversity (PD), which reflects the evolutionary history and genetic relatedness of species, exhibits a complex relationship with species richness and different characteristics (Hähn et al., 2024). Phylogenetic diversity can vary significantly between two communities with equal species richness due to the phylogenetic relatedness of species assemblages (Mittelbach et al., 2007). Higher phylogenetic diversity may facilitate species coexistence by promoting niche differentiation, particularly when species are distantly related. However, this relationship is not guaranteed and must be examined in the context of specific habitats (Ketola, Saarinen & Lindström, 2017). From a dynamic perspective, species richness serves as an immediate indicator of community assembly, whereas phylogenetic diversity reflects long-term evolutionary processes and is more closely linked to the long-term dynamics of community assembly (Jing-Yun et al., 2009). Furthermore, different species occupy distinct positions within phylogenetic clades. Species richness may not fully capture the evolutionary dimensions of biodiversity and represents only one aspect of it (Whitfeld et al., 2014). Therefore, simultaneously assessing taxonomic and phylogenetic diversity may provide a more comprehensive understanding of the evolutionary and ecological values of biodiversity.

The largest subtropical evergreen broadleaf forests in the world are found in East Asia. This region is predicted to experience one of the most significant increases in nitrogen deposition in the future. Furthermore, the net ecosystem carbon exchange rate in these forests exceeds that of tropical rainforests and temperate forests in Asia (Galloway et al., 2004; Yu et al., 2014; Shi et al., 2021). As a result, subtropical forests play a critical role in shaping biodiversity patterns and maintaining ecological security in southern China, warranting increased attention. However, the diversity patterns of plants in subtropical regions are highly complex, influenced by factors such as topography, soil conditions, and climate fluctuations (Liu, Sun & Zhang, 2023). Soil resources also play a pivotal role in the variation of species assemblages, particularly in karst ecosystems where calcareous substrates impose coupled nutrient and water limitations, thereby shaping plant community composition (Michalet et al., 2025). Furthermore, emerging evidence suggests that the interaction between climate and bedrock type is a key driver of ecosystem processes, such as litter decomposition, with effects that cannot be explained by single environmental variables alone. This highlights the importance of explicitly incorporating climate–soil interactions when interpreting biodiversity patterns (Michalet & Liancourt, 2024).

Southwest China is home to numerous typical karst habitats. The mosaic pattern formed by the interweaving of exposed rock formations and shallow soils in these karst landscapes results in spatial heterogeneity in soil ecological functions, which is characterized by rugged terrain, sparse and discontinuous soils, and a lack of surface water. Despite these challenging ecological conditions, karst areas support relatively rich species compositions. Different forest types in karst regions face distinct soil nutrient limitations (Leroux et al., 2017). As core components of soil nutrient cycling, nitrogen (N) and phosphorus (P) play essential roles in regulating forest community composition and plant functional traits. Although nitrogen and phosphorus concentrations are low in karst soils, plant growth in these areas remains co-limited by both elements (Zhang et al., 2019).

Using woody plant survey data from 180 forest-dynamics monitoring plots across Guizhou Province, this study aims to evaluate the effects of climate and soil factors on the diversity patterns of woody plants, considering both their independent and interactive influences. The impacts of key environmental variables on species richness and phylogenetic diversity were quantified using a negative binomial regression model. This study provides a scientific basis for understanding diversity patterns and community assembly in subtropical forests, and offers insights for biodiversity conservation and ecological management in karst regions under global change. Based on this framework, the study addresses the following questions:

(1) How do climate and soil factors individually affect taxonomic and phylogenetic diversity of woody plants?

(2) Do interactions between climate and soil factors enhance the explanatory power of diversity patterns compared to single-factor effects?

(3) Which environmental factors and their interactions play dominant roles in shaping diversity patterns in subtropical karst forests?

## Materials and Methods

### Study sites

The study was conducted in Guizhou Province, the southeastern part of Southwest China, spanning 103°36′–109°35′E longitude and 24°37′–29°13′N latitude. Guizhou lies in the core zone of the karst region of Southwest China, where the exposed karst area accounts for 73% of the total provincial area, ranking first in China (Chen et al., 2016). The landform types are diverse, including plateau canyon, plateau mountain and mountain canyon. The elevation within the province slopes from west to east, and tilts northward, eastward, and southward from the central part. Guizhou Province features a subtropical humid monsoon climate. The annual average temperature across the province is approximately 15 °C, with abundant precipitation. The annual average rainfall ranges from 1,000 to 1,400 mm, and the rainy season is distinct. Influenced by atmospheric circulation, terrain, and other factors, there is significant spatial heterogeneity in the climate across different regions of the province. Such complex landforms and climate conditions have fostered the diversity and complexity of local plants.

**Figure 1:**
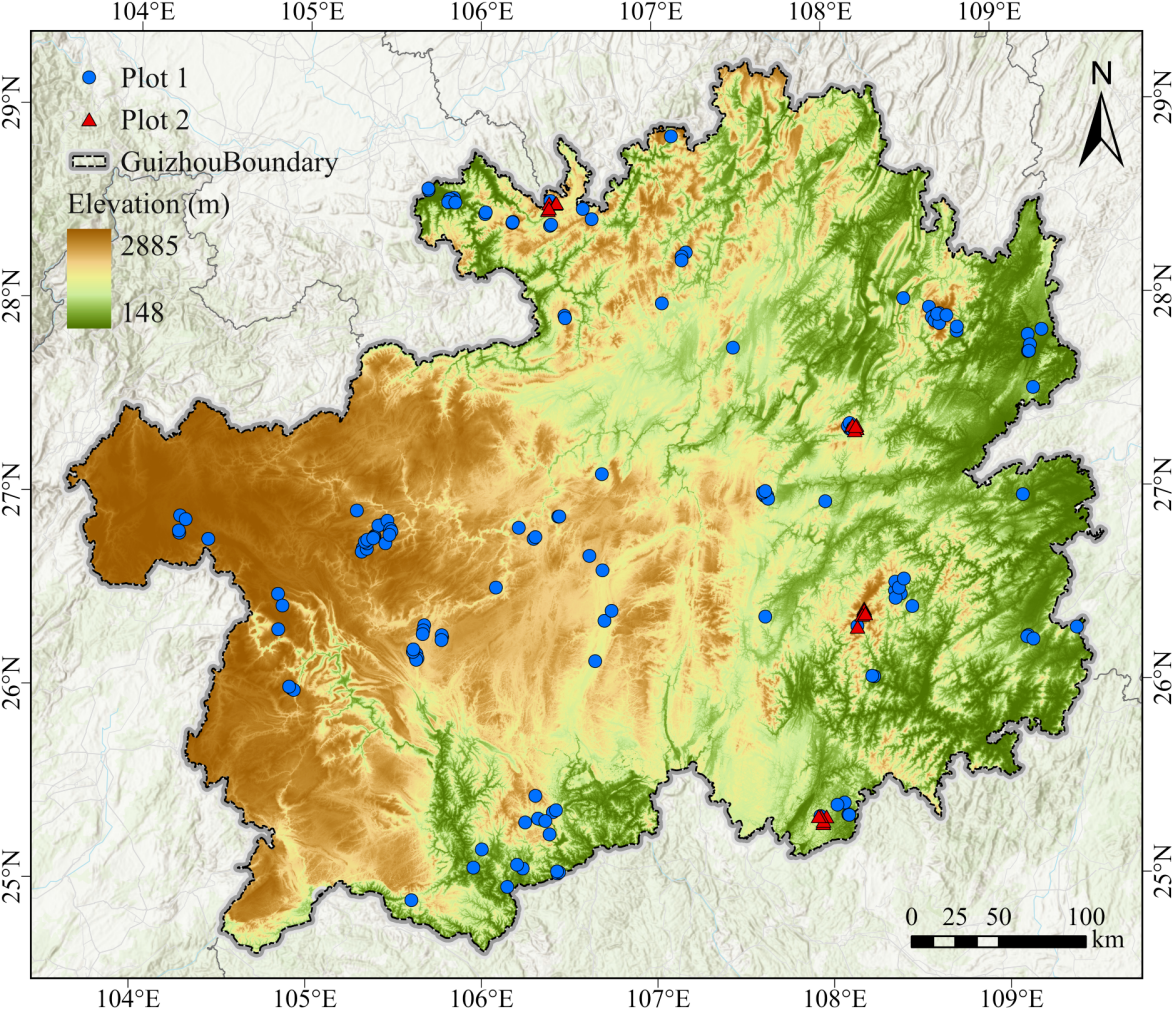
Distribution of woody plant survey plots in Guizhou province. The background color gradient reflects elevation, with brown indicating higher elevations and green indicating lower elevations. Blue solid circles represent historical plots compiled from published monographs (Plot 1), and red solid triangles denote field-surveyed plots (Plot 2).

The survey data of woody plant communities in this study included 180 plots that were derived from two sources (Fig. 1): a part of data was from the standardized plots established by our research team in many nature reserves, national parks, and scenic areas across the province. A total of 20 plots were established by our research team; field plot establishment was approved by the administrative authorities of Maolan National Nature Reserve and other protected areas in Guizhou Province (No.: 2024039). Detailed information on all 180 plots is provided in Table S2, and the remaining data (160 plots) were obtained from monographs such as “*The forest of Guizhou*”, “*Vegetation of Guizhou*”, and some scientific survey reports of nature reserves (the titles of the referenced monographs are shown in Table S1). All plots were surveyed using a consistent quadrat-based sampling method. It recorded the geographical name, longitude and latitude, elevation, plot area, species of woody plants, and species (or relative species) of each species for each plot. For a small number of plots without longitude and latitude information, the corresponding coordinates were retrieved by Google Earth. Since the plot records were collected from different locations at varying times, there are certain variations in plot area. The area ranges from 100 to 20,000 m^2^, with the majority of plots accounting for 82.79% of the total–concentrated between 400 and 800 m^2^. To account for differences in plot size, we log-transformed the sampling area to control for the species–area relationship, thereby ensuring the comparability of diversity estimates across plots. This approach enables us to incorporate area effects appropriately in the model and ensures that estimates of species diversity are comparable across plots of varying sizes.

### Environmental factors

19 worldwide bioclimate factors and elevation data at a 30 arc-second resolution for the study were downloaded from the World Clim database (https://worldclim.org/). Based on the 19 bioclimate factors, potential evapotranspiration (PET) was calculated using the Penman-Monteith equation recommended by the Food and Agriculture Organization of the United Nations (FAO). The exact values of the 20 climate factors (including 19 bioclimate factors and PET) and elevation of 180 plots were obtained using regression kriging with Geostatistical Wizard in ArcMap 10.8.1. Soil data of Guizhou at a 30 arc-second resolution were derived from the Soil Characteristic Dataset of China provided by the Land-Atmosphere Interaction Research Group of Sun Yat-sen University (http://globalchange.bnu.edu.cn/research/data). Because 73% of Guizhou is a karst area with thin soil layers, this study used the topsoil layer (0–0.045 m) of the soil data. Considering the potential collinearity among environmental variables, Pearson’s correlation analysis was conducted for all candidate climate variables. Variables with high collinearity (—r—> 0.7) were excluded, and the remaining variables were retained to reduce multicollinearity and improve model stability. Finally, five climate factors (isothermality (Bio3), temperature seasonality (Bio4), maximum temperature of the warmest month (Bio5), precipitation of the wettest month (Bio13), and potential evapotranspiration (PET)) and ten soil factors (alkali-hydrolyzable nitrogen (AN), bulk density (BD), rock fragment content (GRAV), exchangeable potassium (K), soil porosity (POR), sand (SA), silt (SI), total potassium (TK), total nitrogen (TN), and total phosphorus (TP)) were used in this study (All environmental variables are presented in Table S3).

The plot based basic factors of plots including plot area, longitude, latitude, elevation, landform type, and forest type were used in this study. Landform types included karst and non-karst landforms. Based on the forest classification criteria of the World Wide Fund for Nature (WWF), the plots were classified into six forest types: evergreen broad-leaved forest (EBF), deciduous-evergreen broad-leaved mixed forest (DEBMF), deciduous broad-leaved forest (DBF), coniferous-broad-leaved mixed forest (CBMF), evergreen coniferous forest (ECF), and montane forest (MF).

### Calculating taxonomic and Faith’s phylogenetic diversity

In this study, the species richness (SR) and phylogenetic diversity (PD) were used to characterize the taxonomic and phylogenetic diversity of each community, respectively. The Faith’s phylogenetic diversity quantified the phylogenetic diversity of each community by calculating the sum of the phylogenetic branch lengths of all species in a plot (Faith, 1992). This index not only quantifies the evolutionary divergence of the community but also profoundly reflects the evolutionary history and genetic relatedness of species. A total of 610 woody plant species were recorded, belonging to 239 genera and 81 families, after integrating and verifying the community data of all plant survey plots. The top five species with the widest distribution in all plots are *Quercus fabri, Quercus glauca*, *Liquidambar formosana*, *Rhododendron simsii* and *Cunninghamia lanceolata*, respectively.

We generated a phylogenetic tree for 610 species across 180 plots using the “V. PhyloMaker” package (Jin & Qian, 2023), with all 610 species successfully incorporated into the tree. Using a time-calibrated maximum likelihood estimation, the “V. PhyloMaker” package generated two species-level mega-phylogenetic trees with the dataset of more than 70,000 vascular plant species based on the GenBank taxa with a backbone provided by Open Tree of Life version 9.1 (GBOTB) from Smith & Brown (2018) and the phylogenetic information from Zanne et al. (2014).

### Modeling procedure

A significant overdispersion in species richness was revealed among 180 plots. By incorporating a dispersion parameter, the negative binomial regression model can flexibly address data overdispersion, avoiding the estimation bias caused by violations of model assumptions. Therefore, the negative binomial regression model was used in this study for analyzing the relationships between species richness/Faith’s phylogenetic diversity and climate and soil factors. Model selection was performed using a stepwise procedure based on the Akaike Information Criterion (AIC), implemented through the “step ()” function in R. The AIC evaluates models by balancing goodness-of-fit and model complexity, thereby reducing the risk of overfitting caused by excessive parameters. In addition, a log link function, which is the default in negative binomial generalized linear models, was adopted in this study. The modeling procedure was constructed through the following steps:

This study established the following modeling framework: species richness and Faith’s phylogenetic diversity were modeled separately as response variables. For each response variable, five models with different combinations of independent variables were constructed, including basic plot variables, climate variables, soil variables, their combined effects, and climate–soil interactions. This design allowed us to quantify and compare the relative contributions of different variable groups to the variation in the two diversity indices (Dattaraja et al., 2018; Shi et al., 2024).

(a) Combination of basic factors: only plot-based basic factors were included, whereas climate and soil factors were excluded.

(b) Combination of climate factors: plot-based basic factors and climate factors were included, whereas soil factors were excluded.

(c) Combination of soil factors: plot-based basic factors and soil factors were included, whereas climate factors were excluded.

(d) Combination of all factors: plot-based basic factors, climate factors, and soil factors were all included.

(e) Combination of climate–soil interactions: plot-based basic factors, climate factors, soil factors, and the interactions between climate and soil factors were included.

To reduce the influence of spatial clustering and potential non-independence among nearby plots, a fishnet grid with a size of 2 km × 2 km was applied to cover the study area. If more than one plot occurred within a grid cell, only one plot was randomly selected in each iteration. This spatial thinning approach ensures that at most one plot is included per grid cell, thereby reducing redundancy and spatial autocorrelation among neighboring observations. A total of 119 out of 180 plots were selected for modeling in each iteration. This random selection procedure was repeated 999 times, and model results were averaged across all iterations to improve the robustness of parameter estimates and reduce sensitivity to spatial sampling bias. Model complexity was controlled independently using an Akaike Information Criterion (AIC)-based model selection procedure. For each combination of variables, the same set of predictor variables was used across all 999 repetitions, while only the sampled plots varied due to random spatial subsampling. The mean and standard deviation across the 999 repetitions were calculated to summarize the variability introduced by the spatial subsampling procedure. For the five resulting models, we further performed variance decomposition. By comparing the increments in the relative effects (%R^2^) of each variable component within each variable combination across nested models, the total explained variance of each model was decomposed into the independent contributions of different variable groups.

The data processing was primarily conducted using R software (version 4.4.3; R Core Team, 2025). The “MASS” package was used for negative binomial model analysis; the “V. PhyloMaker”, “ape”, and “picante” packages were used for Faith’s phylogenetic diversity analysis; the “mgcv” and “gstat” packages were used for interpolation analysis by a regression kriging method. Additionally, ArcMap (version 10.8.1) was used to process the data.

## Results

### The effects of climate and soil on species richness

**Figure 2:**
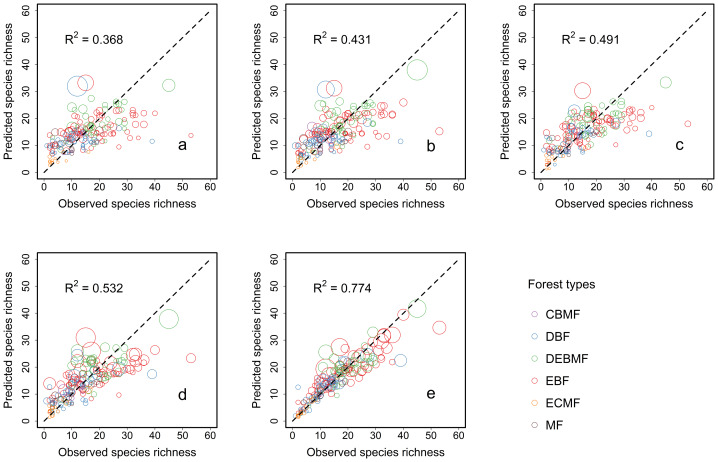
Differences between the observed species richness and the predicted species richness predicted by negative binomial regression model under different variable combinations. The *x* and *y* coordinates of the center of each circle are the observed species richness and the average value of predicted species richness of 999 replicates, respectively. The radius of each circle is the standard deviation of predicted species richness of 999 replicates. Variable combinations used in the figure: (A) the combination of basic factors; (B) the combination of climate factors; (C) the combination of soil factors; (D) the combination of all factors; (E) the combination of climate–soil interactions. Forest types: evergreen broad-leaved forest (EBF), deciduous-evergreen broad-leaved mixed forest (DEBMF), deciduous broad-leaved forest (DBF), coniferous-broad-leaved mixed forest (CBMF), evergreen coniferous forest (ECF), and montane forest (MF).

**Figure 3:**
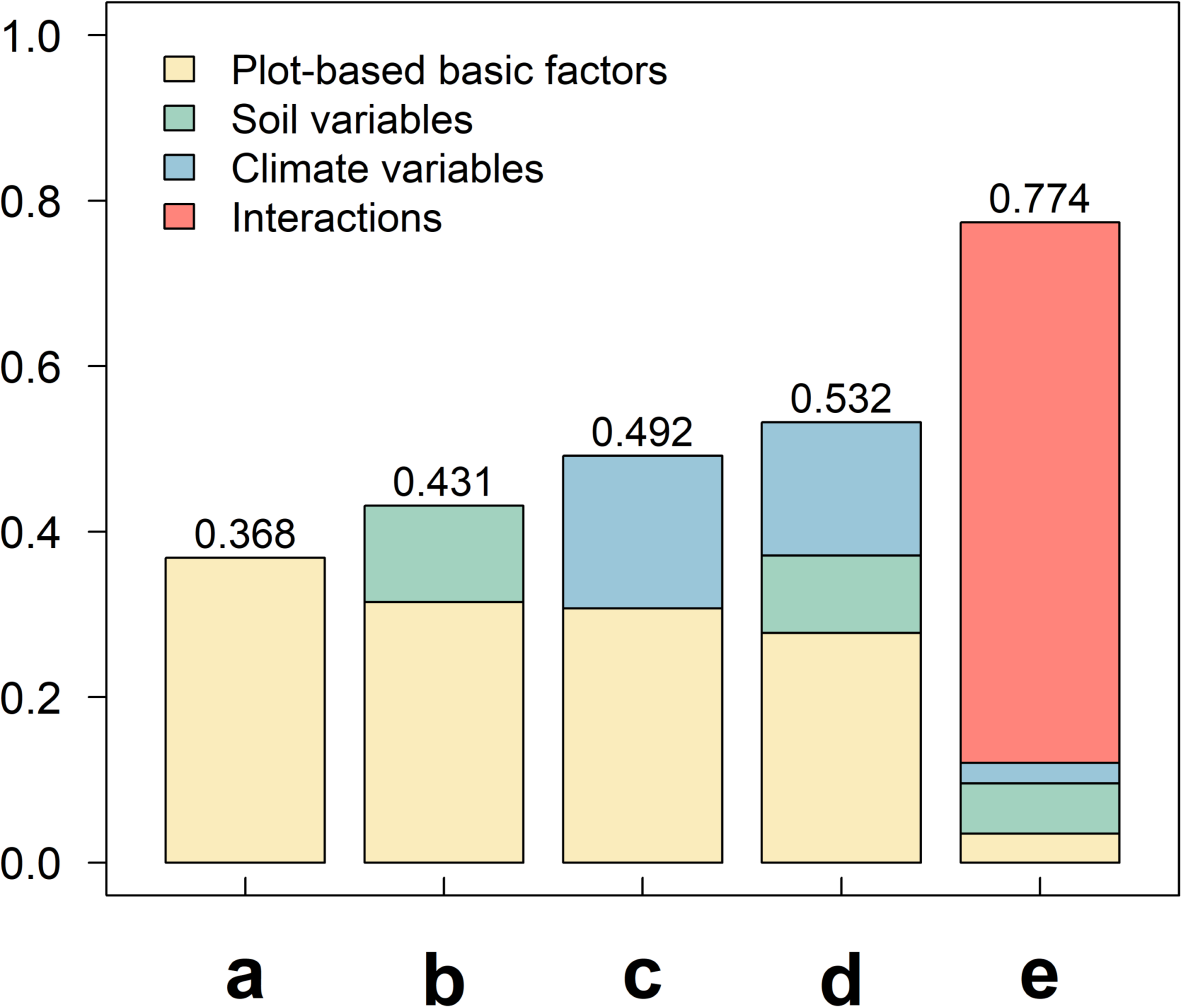
The proportion of variation in species richness explained by different variable combinations. Variable combinations used in the figure: (A) the combination of basic factors; (B) the combination of climate factors; (C) the combination of soil factors; (D) the combination of all factors; (E) the combination of climate–soil interactions.

The modeling results showed that the proportion of the variation in species richness explained solely by plot-based basic variables was low (*R*^2^ = 0.368, Fig. 2A). The proportion of the variation explained by the combination of soil factors (*R*^2^ = 0.431, Fig. 2B) and the combination of climate factors (*R*^2^ = 0.491, Fig. 2C) was higher, separately. It demonstrated that changes in soil and climate factors both influence species richness, and that climate factors exert a slightly stronger effect when both are taken into account (Figs. 2C and 2D), the addition of these factors further enhanced the model’s explanatory power (*R*^2^ = 0.532). However, after considering the effect of interactions between climate and soil factors (Fig. 2E), the model’s explanatory power increased substantially, with the R^2^ value reaching as high as 0.774, representing a substantial increase of 45.49% comparing to the power explained by the combination of all factors (Fig. 3). These results indicated that the impact of climate–soil interactions is significantly greater than that of single-type environmental factors, and such interactions can more comprehensively explain the variation in species richness.

### The impacts of climate and soil factors on Faith’s phylogenetic diversity

**Figure 4:**
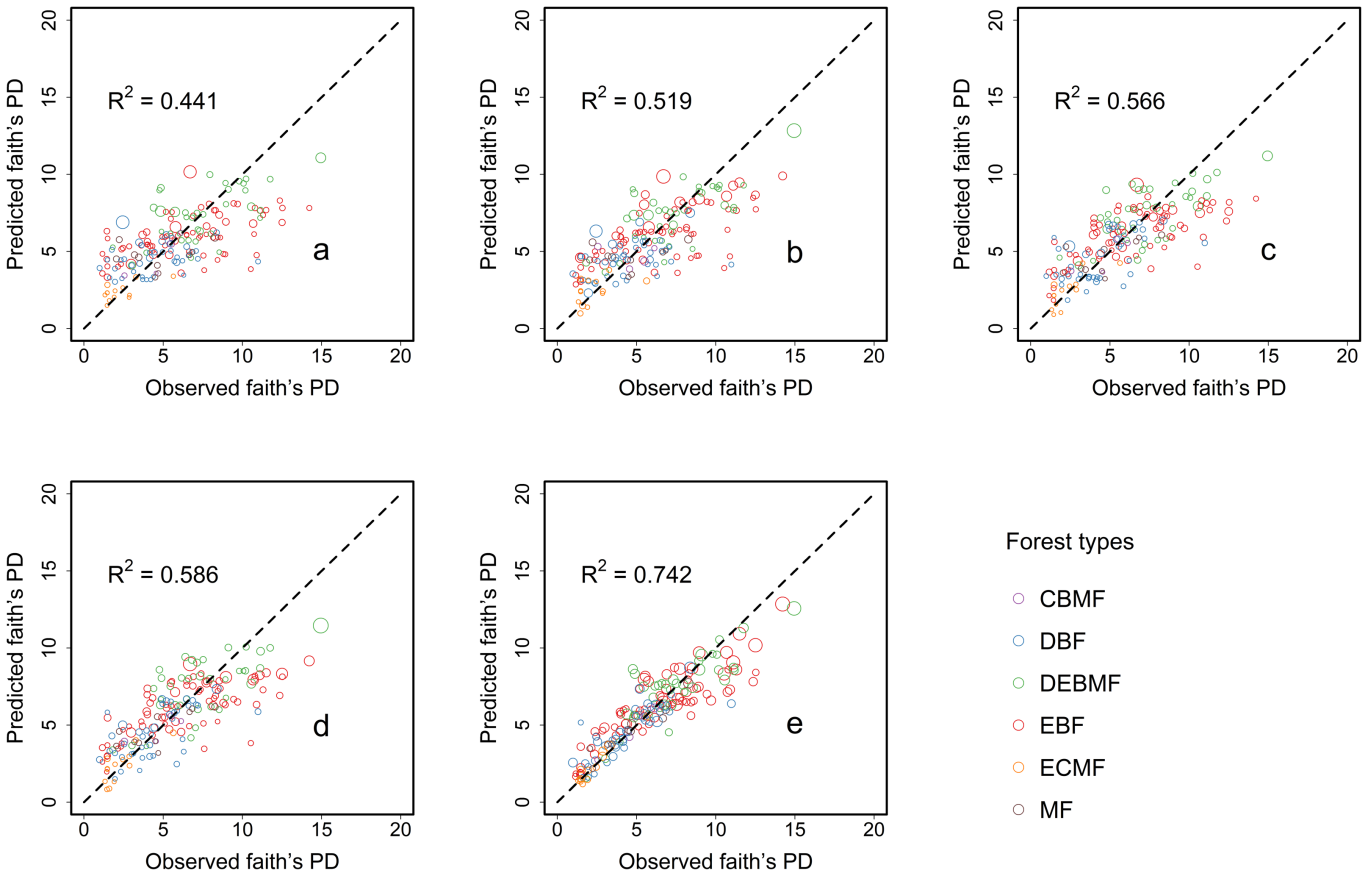
Differences between the observed Faith’s phylogenetic diversity and the predicted species richness predicted by negative binomial regression model under different variable combinations. The *x* and *y* coordinates of the center of each circle are the observed Faith’s phylogenetic diversity and the average value of predicted Faith’s phylogenetic diversity of 999 replicates, respectively. The radius of each circle is the standard deviation of predicted faith’s phylogenetic diversity of 999 replicates. Variable combinations used in the figure: (A) the combination of basic factors; (B) the combination of climate factors; (C) the combination of soil factors; (D) the combination of all factors; (E) the combination of climate–soil interactions. Forest types: evergreen broad-leaved forest (EBF), deciduous-evergreen broad-leaved mixed forest (DEBMF), deciduous broad-leaved forest (DBF), coniferous-broad-leaved mixed forest (CBMF), evergreen coniferous forest (ECF), and montane forest (MF).

**Figure 5:**
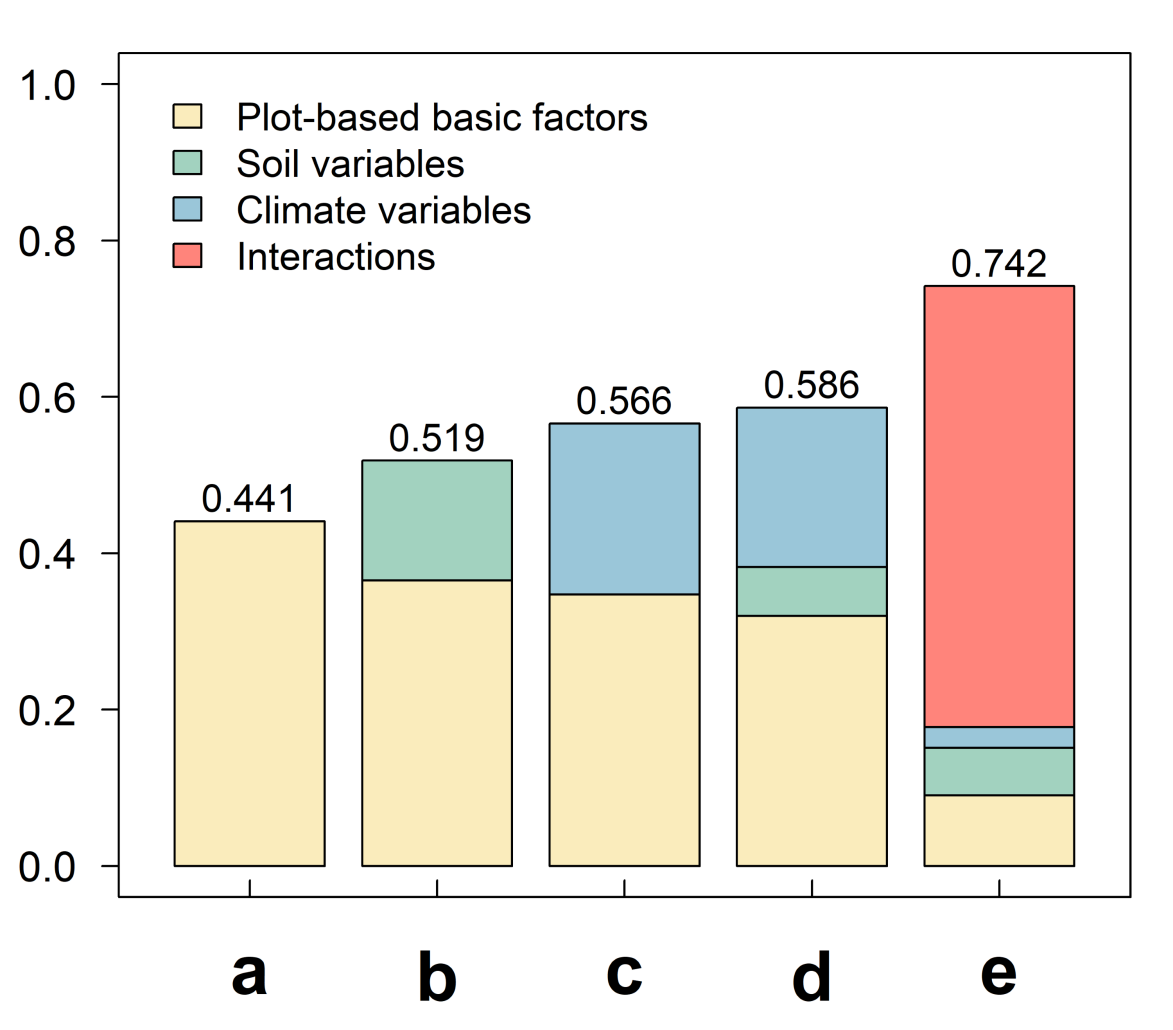
The proportion of variation in Faith’s phylogenetic diversity explained by different variable combinations. Variable combinations used in the figure: (A) the combination of basic factors; (B) the combination of climate factors; (C) the combination of soil factors; (D) the combination of all factors; (E) the combination of climate–soil interactions.

Similar to the modeling results in species richness, the modeling results for Faith’s phylogenetic diversity showed that the proportion of the variation in Faith’s phylogenetic diversity explained solely by plot-based basic variables was low (*R*^2^ = 0.441, Fig. 4A). The proportion of the variation explained by the combination of soil factors (*R*^2^ = 0.519, Fig. 4B) and the combination of climate factors (*R*^2^ = 0.566, Fig. 4C) has improved, separately. It demonstrated that changes in both soil and climate factors affect Faith’s phylogenetic diversity. Climate factors had a slightly stronger impact than soil factors. When both factors were considered simultaneously (Fig. 4D), the addition of these factors further enhanced the model’s explanatory power (*R*^2^ = 0.586). However, after considering the effect of interactions between climate and soil factors (Fig. 4E), the model’s explanatory power increased by 26.62%, with the R^2^ value reaching as high as 0.742, representing a substantial increase compared to the power explained by the combination of all factors (Fig. 5). These results indicated that the impact of climate-soil interactions is significantly greater than that of single-type environmental factors, and such interactions can more comprehensively explain the variation in Faith’s phylogenetic diversity.

### Driving factors for species richness and Faith’s phylogenetic diversity

**Figure 6:**
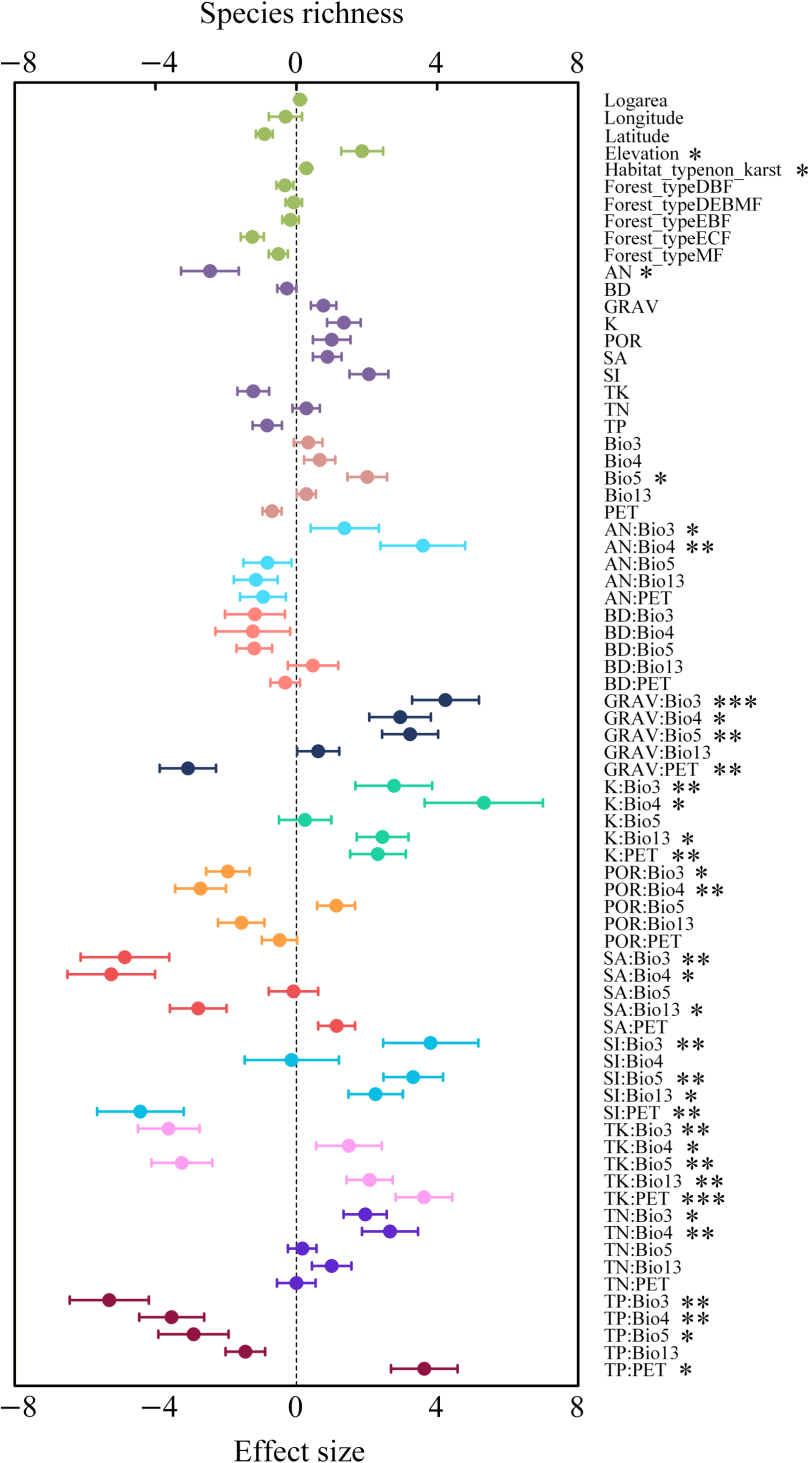
Average relative effects and the relevant average 95% confidence intervals of plot-based basic factors, climate factors, soil factors, and the interactions between climate and soil factors on species richness. The *p*-values of the predictor variables are indicated as follows: *p < 0.05; **p < 0.01; ***p < 0.001.

**Figure 7:**
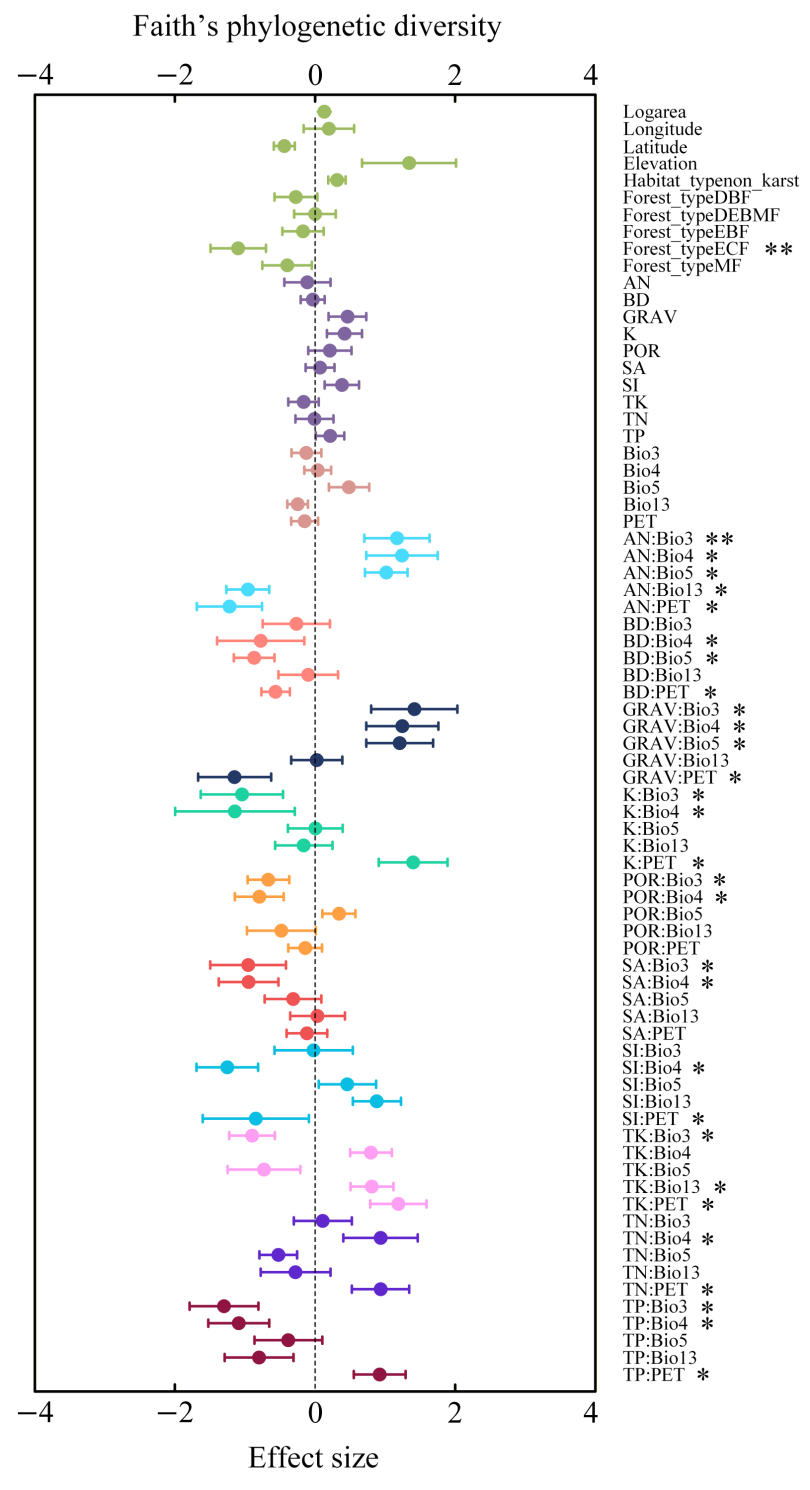
Average relative effects and the relevant average 95% confidence intervals of plot-based basic factors, climate factors, soil factors, and the interactions between climate and soil factors on Faith’s phylogenetic diversity. The *p*-values of the predictor variables are indicated as follows: *p < 0.05; **p < 0.01; ***p < 0.001.

The effects of individual environmental factors on species richness (SR) and Faith’s phylogenetic diversity (Faith’s PD) vary substantially (Figs. 6 and 7). Overall, most single soil or climate variables exhibited limited direct effects on SR and Faith’s PD; however, their interactions often exerted much stronger influences. For example, interactions between rock fragment content (GRAV) and some climate factors such as isothermality (Bio3) and temperature seasonality (Bio4) exerted significant effects on both Faith’s PD and SR. In general, soil nutrients such as alkali-hydrolyzable nitrogen (AN), total nitrogen (TN), exchangeable potassium (K), and rock fragment content (GRAV), acted as key positive predictors of Faith’s PD when interacting with temperature-related variables (such as Bio3, Bio5). The interaction between exchangeable potassium and temperature seasonality (K: Bio4) yielded the strongest positive impact on SR, followed by AN: Bio4 and GRAV: Bio3. This pattern indicates that soil potassium availability, nitrogen status, and coarse-fragment structure become particularly influential for species richness when modulated by key temperature dynamics. At the same time, several climate–soil interactions negatively affected species richness. For instance, SA: Bio4 and TP: Bio3 showed significant suppressive effects. Additionally, interactions involving total potassium (TK) and soil porosity (POR) with temperature-related variables (Bio2, Bio3) were associated with reductions in SR, although their magnitudes varied.

## Discussion

Many previous studies have demonstrated that climate and soil factors influence plant community assembly. This influence primarily drives plant community assembly through direct physiological filtering (Veldkornet & Adams, 2025), resource allocation (*e.g.*, soil nutrients) (Inague, Zwiener & Marques, 2021; Tetemke et al., 2021; Chen et al., 2025), or evolutionary constraints (Wicke & Fischer, 2018). However, some factors often have a direct or indirect influence on plant communities through the interactions with other factors. For instance, soil properties are key drivers of community structure, but soil moisture content, one of the soil properties, which was directly influenced by local precipitation, filters the certain dominant species in the community, resulting in a variation of plant diversity and density (Guo et al., 2022). Additionally, climate factors can modulate the physical and chemical properties of soil through the processes such as weathering, leaching, and organic matter accumulation, which in turn affect plant growth (Schnoor, Bruun & Olsson, 2015; Wang, Wang & Gao, 2022). These findings strongly indicated that the combined effects of factors can effectively deepen their impacts on community diversity. Our research also supported this conclusion: the dominant driving force driving the patterns of species richness and phylogenetic diversity of woody plants in Guizhou was the interactions of climate and soil factors.

Comparing to the non-karst areas, karst areas are more fragile and variable, and their ecosystems are extremely sensitive to environmental changes and disturbances. The karst habitat conditions is spatiotemporally heterogeneous (Yang et al., 2015), which affect the formation, distribution, and physical-chemical properties of soil, altering the distribution of water and heat, and thereby influence the maintenance of plant diversity in karst regions (Zhang et al., 2019). Habitat heterogeneity is the core foundation for the formation and evolution of biodiversity. In karst areas, the complex topographic conditions (*e.g.*, diverse landforms such as peak clusters, depressions, and canyons) further exert more fine-grained effects on plant communities through the regulation of microenvironments. For instance, the water use efficiency of evergreen species varies to adapt to the local microenvironment, thereby adjusting the degree of niche overlap among different species, and ultimately resulting in the composition and dynamic changes of regional species diversity (Inague, Zwiener & Marques, 2021).

Soils in karst landscapes exhibit pronounced spatial heterogeneity in nutrient availability. The interactions between soil properties and climatic conditions frequently regulate the accessibility of key limiting resources, particularly phosphorus and water (He & Dijkstra, 2014; Chen et al., 2025). More specifically, climate conditions (*e.g.*, temperature variability and precipitation regimes) act as regulators that modify the rate of nutrient cycling, microbial activity, and soil moisture dynamics, thereby determining whether soil nutrients can be effectively utilized by plants. These interactions shape patterns of phylogenetic differentiation and species turnover within plant communities. In this study, interactions involving alkali-hydrolyzable nitrogen (AN), total phosphorus (TP), total potassium (TK), as well as soil physical properties such as rock fragment content (GRAV) and soil porosity (POR), emerged as the principal drivers of variation in both species richness and phylogenetic diversity. Temperature-related factors—diurnal temperature range and temperature seasonality—showed the strongest interactive effects with soil properties on woody plant diversity. This indicates that temperature dynamics do not directly determine diversity, but instead modulate the strength and direction of soil effects on plant communities. On the other hand, precipitation exerted relatively weak direct effects. However, its interaction with soil physicochemical properties modulates water availability, thereby exerting an influence on community composition in microhabitats where water limitation is critical (Inague, Zwiener & Marques, 2021; Tetemke et al., 2021). In such cases, precipitation mainly operates as a controlling factor of soil water retention and release processes rather than as an independent driver of diversity.

Nitrogen (AN, TN) and potassium (K, TK) made substantial contributions. In particular, the interaction between exchangeable potassium and climatic conditions had a significant effect on species richness, this phenomenon is likely explained by the central role of potassium in governing plant growth, stomatal function, and stress tolerance. As a key ion governing stomatal opening and closure, the bioavailability of potassium can amplify the filtering effects of climate in regions characterized by seasonal drought or pronounced precipitation variability (Sardans & Peñuelas, 2015). In other words, climate variability determines the degree to which potassium becomes a limiting or facilitating resource, thereby strengthening its ecological role under stressful conditions. Species with higher potassium-use efficiency are better able to maintain physiological homeostasis under water stress through more effective stomatal regulation. Such physiological advantages confer stronger competitive performance, ultimately influencing community composition and the spatial distribution of dominant species. This result further demonstrates that soil nutrients influence diversity not in isolation, but through climate-dependent resource limitation mechanisms.

The strong influence of rock fragment content, bulk density, and soil porosity is closely linked to karst-specific soil formation (Laliberté et al., 2013). Karst soils originate from carbonate rock weathering and are typically shallow, discontinuous, and rich in coarse fragments, with a low volume of fine materials and extensive macropore development. These characteristics reduce water-holding capacity and nutrient retention (Zhu et al., 2017), and intensify nutrient leaching under the high-precipitation condition in Guizhou. Under such conditions, climate factors—especially precipitation and temperature fluctuations—including isothermality (Bio3), temperature seasonality (Bio4), maximum temperature of the warmest month (Bio5), and precipitation of the wettest month (Bio13), which respectively characterize temperature stability, temporal variability, thermal extremes, and peak water input—interact with soil structure to jointly control water availability and nutrient loss processes. At broad scales, resource scarcity and drought stress remain dominant forces for shaping the taxonomic and phylogenetic diversity pattern. However, microhabitats with low rock fragment content, reduced bulk density, and high porosity tend to support great water and nutrient availability, potentially resulting in high local species richness at fine scale. Soil conditions characterized by high rock fragment content, elevated bulk density, and low soil porosity enhance environmental filtering by favoring species with conservative traits adapted to drought and nutrient-poor substrates. This selective pressure promotes phylogenetic clustering (Cai et al., 2021). Here, climate variability further intensifies this filtering process by increasing resource instability, thereby strengthening the selective advantage of stress-tolerant lineages. Nevertheless, some ancient or specialized lineages possess long-evolved adaptations—such as deep rooting systems or lithophytic growth forms—that enable their persistence under severe resource constraints (Zotz, Hietz & Schmidt, 2001), preventing a strict decline in phylogenetic diversity with species richness. Overall, soil-climate interactions jointly construct a strong environmental filtering framework, where resource limitation, hydrological variability, and nutrient imbalance collectively shape diversity patterns.

The proportion of variation explained in phylogenetic diversity was higher than that in species richness for the first four combinations of factors, but became lower under the climate–soil interactions combination (Shi et al., 2024). This pattern suggests that phylogenetic diversity is more strongly associated with single environmental gradients, whereas species richness is more sensitive to the combined effects of climate and soil factors. A plausible explanation is that the phylogenetic structure of a community reflects the integrated influences of ecological interactions, environmental filtering, and historical evolutionary processes (Yan, Yang & Tang, 2013). In southern China, patterns of phylogenetic diversity are largely shaped by long-term speciation and extinction dynamics, as well as geological history—for example, the uplift of the Qinghai–Tibet Plateau, which elevated the carbonate bedrock crust in Guizhou, and Quaternary glaciations, which indirectly influenced karst development by stripping weathered carbonate layers and generating extensive periglacial landforms.

In addition, many endemic species in karst regions exhibit strong niche conservatism, causing their phylogenetic relationships to respond more slowly to short-term environmental changes than species richness, which can adjust rapidly through processes such as species migration or local extinction. This further indicates that phylogenetic diversity tends to capture long-term environmental filtering signals and shows relatively weaker responsiveness under complex multi-factor interactions. Species richness, as an immediate indicator of community composition, tends to be more sensitive to environmental variation. In contrast, phylogenetic diversity—representing deeper evolutionary divergence and the genetic basis for adaptive responses—changes more slowly when environmental conditions shift.

Previous studies have shown that while individual climate or soil variables explain only a limited proportion of variation in species richness, their interactions can account for a substantially larger fraction of explained variance (Shi et al., 2024). Moreover, phylogenetic diversity generally exhibits stronger correlations with environmental variables than species richness, indicating its higher sensitivity to environmental filtering. Therefore, the reduction in explanatory power for phylogenetic diversity under climate–soil interaction scenarios may reflect the increasing influence of stochastic processes or multi-factor constraints, whereas species richness is more directly regulated by resource availability mediated through climate–soil coupling (He & Dijkstra, 2014).

The area of 180 plots used in this study varied greatly, ranging from 100 to 20,000 m^2^. There would be inherent differences in the observed species between small and large plots due to the sampling size. The omission of rare species and the underestimation of true diversity would likely occur. Moreover, the negative binomial regression models used in this study showed better goodness-of-fit when interactions were included. A potential reason for this may be the substantial increase in the number of variables; the new interactions partition the explanatory space, thereby diluting the contribution of individual factors. Finally, this study was focused on Guizhou, a subtropical region in Southwest China dominated by karst topography. The relationships and gradient variations of taxonomic diversity and phylogenetic diversity with multiple environmental factors at larger scales remain to be explored.

It should be noted that the variability estimated in this study is derived from the spatial subsampling procedure, which primarily reflects the influence of spatial clustering and sample selection. Although this approach improves robustness under spatial constraints, it does not fully capture the overall sampling variability. More rigorous resampling strategies, such as bootstrap methods that resample all plots with replacement, could provide a more comprehensive estimation of uncertainty and should be considered in future studies.

## Conclusion

This study focused on the impacts of climate, soil factors, and the interaction on the patterns of taxonomic and phylogenetic diversity of woody plants in Guizhou. The results showed that the performance of model with the combination of all factors and interactions between climate and soil factors was significantly higher than the models with any other combination of factors. It revealed that the interactions between climate and soil are the dominant driving force to taxonomic and phylogenetic diversity patterns. We argue that it is necessary to considering the interactions of different type of factors for the further understanding of environmental filtering in shaping the diversity patterns of woody plants.

##  Supplemental Information

10.7717/peerj.21365/supp-1 Supplemental Information 1 Bibliography of references for quadrat data of woody plants in Guizhou

10.7717/peerj.21365/supp-2 Supplemental Information 2 Study plot information

10.7717/peerj.21365/supp-3 Supplemental Information 3 Environmental variables

## Additional Information and Declarations

### Field Study Permissions

The following information was supplied relating to field study approvals (i.e., approving body and any reference numbers): Field plot establishment was approved by the Administration Bureau of Maolan National Nature Reserve, Guizhou Province (No.: 2024039).

### Data Availability

The following information was supplied regarding data availability: The data and analysis code are available at GitHub and Zenodo: – https://github.com/ZJ20000909/R_code.git. – ZJ20000909. (2025). ZJ20000909/R_code: R_code (v1.0.0). Zenodo. https://doi.org/10.5281/zenodo.17600056.
