# Supplementary material for: The interactions between climate and soil factors influence the taxonomic and phylogenetic diversity of woody plants in the subtropical karst region
Source: PeerJ. 2026 May 22;14:e21365. doi: 10.7717/peerj.21365 (PMC13200658; doi:10.7717/peerj.21365)
Supplement: Supplemental Information 1 [file peerj-14-21365-s001.docx]

Supplementary Table 1. Bibliography of References for Quadrat Data of Woody Plants in Guizhou

| Title of Monograph | Editor-in-Chief | Publisher/Source |
| --- | --- | --- |
| The forests of Guizhou | Zhengxian Zhou | Guizhou Science and Technology Publishing House |
| Vegetation of Guizhou | Weilian Huang, Yulin Tu, Long Yang | Guizhou People's Publishing House |
| Scientific Survey and Research of the Guizhou Xishui Subtropical Evergreen Broad-Leaved Forest National Nature Reserve | Yang Luo, Lang Liu, Rongyuan Yang | Guizhou Science and Technology Publishing House |
| Scientific Survey Collection of Nangong Nature Reserve | Huahai Zhang, Guizhou Forestry Department | Guizhou Science and Technology Publishing House |
| Scientific Survey Collection of Laoshechong Nature Reserve | Huahai Zhang, Guizhou Forestry Department | Guizhou Science and Technology Publishing House |
| Scientific Survey Collection of the Guizhou Baili Azalea Forest Area | Zhenye Liu, Guizhou Association for Science and Technology | Guizhou Association for Science and Technology |
| Ecology Research on Karst Forest—III | Shouqian Zhu | Guizhou Science and Technology Publishing House |
| Scientific Survey and Research on Nayong Nature Reserve for Davidia involucratain Guizhou, China​ | Lunxiu Deng, Jingcheng Ran, Jinwen Shang | China Forestry Publishing House |
| Guizhou Wangmo Cycas Nature Reserve | Yang Luo, Lang Liu | Guizhou Science and Technology Publishing House |
| Biodiversity of the Guizhou Chishui Alsophila National Nature Reserve | Hongping Deng | Science Press |
| Scientific Survey of the Zhujiashan Nature Reserve in Guizhou | Guizhou Forestry Department | China Forestry Publishing House |
| Scientific Survey of the Fanjingshan Mountain Preserve, Guizhou Province, China | Editorial Committee of the Scientific Survey of Fanjingshan, Guizhou | China Environmental Science Press |
| Scientific Survey of the Fodingshan Nature Reserve in Guizhou​ | Guizhou Forestry Department | China Forestry Publishing House |
| CERN Long-term Biological Sample Plot Background and Plant Taxonomic Specimen Baseline Dataset |  | National Ecosystem Science Data Center |
